# Factors Associated with Halitosis in White-Collar Employees in Shanghai, China

**DOI:** 10.1371/journal.pone.0155592

**Published:** 2016-05-17

**Authors:** Xi Chen, Yu Zhang, Hai-Xia Lu, Xi-Ping Feng

**Affiliations:** Department of Preventive Dentistry, Ninth People’s Hospital, Shanghai Jiao Tong University, School of Medicine, Shanghai Key Laboratory of Stomatology, Shanghai, China; National Institute of Agronomic Research, FRANCE

## Abstract

**Objective:**

To investigate the factors associated with halitosis in Chinese white-collar employees.

**Materials and Methods:**

Subjects in three randomly selected office buildings in Shanghai, China, were enrolled in this cross-sectional study using cluster random sampling. Oral malodor was assessed by measuring volatile sulfur compounds (VSCs) with a portable sulfide monitor. Subjects’ oral health, including dental caries, periodontal status, and tongue coating, was evaluated clinically. A questionnaire was used to obtain information about participants’ demographic characteristics, oral hygiene habits, and health behaviors.

**Results:**

Of the 805 subjects invited to participate in this study, 720 were enrolled (89.4% response rate). Data from these subjects were used for statistical analyses. The prevalence of halitosis was 33.2%. In the final regression model, halitosis was significantly related to tongue coating thickness, periodontal pocket depth, no food consumption within 2 hours prior to oral examination, and less intake frequency of sweet foods.

**Conclusions:**

In this Chinese white-collar population, tongue coating and periodontal disease were associated with halitosis. Oral hygiene education should be provided at the population level to encourage the maintenance of oral health and fresh breath. Consumption of sweet foods may reduce VSC production, although this finding requires further investigation.

## Introduction

The term “halitosis” refers to an unpleasant or offensive odor emanating from the mouth, regardless of whether its source is oral or non-oral[1–4]. Other terms used to describe this health condition are bad breath, foul breath, breath odor, oral malodor, *foetor ex ore*, and offensive breath[3]. The main chemical components of halitosis are volatile sulfur compounds (VSCs), such as hydrogen sulfide, methyl mercaptan, and dimethyl sulfide. Other substances known to contribute to this condition are ammonia, amines, putrescine, cadaverine, and butyric acid[2,5,6].

Most cases of halitosis (80–90%) originate in the oral cavity as a result of proteolytic degradation by anaerobic Gram-negative oral bacteria of various sulfur-containing substrates in saliva, epithelial cells, blood, and food debris[2,7]. The dorsum of the tongue and periodontal pockets play important roles in VSC formation[8]. Non-oral sources of halitosis include upper respiratory problems, some metabolic disorders, and certain gastrointestinal tract disturbances[9,10]. The reported prevalence of oral malodor has ranged from 14% to >50% in different populations, although previous studies have used different assessment methods and cutoff points for VSC values[3,11–20]. The prevalence of and/or factors related to halitosis have been examined in diverse segments of populations, such as young mothers, elderly people, army recruits, high-school students, and adolescents[13,16,17,19,20].

Compared to other workers, white-collar employees, including administrators, office workers, technicians, and commercial staff, may have more opportunities for interpersonal communication and, as a result, must pay more attention to their personal appearance. Many studies have examined aspects of health, such as mental health problems, sleep quality, and other illnesses, among this population [21–23]. However, no study has examined the prevalence of or factors related to halitosis in white-collar workers. Therefore, the aim of this study was to explore the prevalence of and factors associated with halitosis in white-collar employees using a self-administered questionnaire. In addition to oral factors potentially contributing to halitosis, we also evaluated the influence of factors such as diet, lifestyle habits, systematic discomfort, and disease.

## Materials and Methods

The study was in compliance with Strengthening the reporting of observational studies in epidemiology (STROBE) statement guidelines.

### Study population

This cross-sectional study was conducted in three randomly selected districts among the six districts with concentrations of office buildings in Shanghai, China. One office building in each district and two departments in each building were selected using cluster random sampling. All staff members in these departments were verbally invited to participate in the study by department directors who had been informed of the purpose and structure of the study. The sample size was calculated on the basis of a prevalence of halitosis of 20% in the general Chinese population, as determined by Halimter [12]. The minimum sample size for the study was determined to be 512 participants; allowing for a 15% non-response rate, a sample of at least 590 participants was sought. The Ethics Committee of the Ninth People’s Hospital, School of Medicine, Shanghai Jiao Tong University approved this study. All participants provided written informed consent prior to examination and questionnaire administration. The study was performed in accordance with the principles of the Helsinki Declaration.

### Examination procedure

In each office building, we rented a room for several days for examination purposes. Subjects were asked to complete the questionnaire, and then oral malodor was assessed. Clinical oral examination was conducted with each subject seated in a portable dental chair. Three dentists were involved in the investigation, each of whom was responsible for one procedure. The examination procedure, which required about 2 hours for each subject, was conducted during the working hours of each office building. Subjects were not given specific instructions (e.g., to brush) or prohibitions (e.g., to not eat).

### Oral malodor assessment

Oral malodor was assessed with a Halimeter (Interscan, Chatsworth, CA, USA) by measuring VSCs originating from the oral cavity. Each subject was asked to close his/her mouth for at least 1 minute. He/she was then instructed to hold a disposable tube above the posterior tongue dorsum, with the mouth slightly open while breathing through the nose. The peak value [in parts per billion (ppb)] displayed by the Halimeter was recorded. A VSC value of 110 ppb or greater was considered to indicate the presence of halitosis, according to the manufacturer’s instructions [24].

### Clinical oral examination

A trained and licensed dentist assessed participants’ dental caries status, gingival inflammation, periodontal status, plaque accumulation, and tongue coating. Oral examinations were conducted after oral malodor assessment to avoid the potential effect of possible gingival bleeding after probing on the accuracy of the latter. Decayed, missing, and filled tooth (DMFT) and decayed, missing, and filled surface (DMFS) indices were calculated. Dental caries status was assessed using the visual-tactile criteria proposed by the World Health Organization [25] for the diagnosis of pit-and-fissure and smooth-surface lesions. Gingival inflammation and plaque accumulation were examined using the gingival index (GI) and plaque index (PLI) at four sites (mesial, distal, buccal, and lingual) on six index teeth (#3, #8, #11, #19, #25, and #28) [26–28]. Periodontal probing depth (PPD) was measured at four sites on each tooth using a scaled periodontal probe. Tongue coating was evaluated according to the area covered (0 = none, 1 = <1/3 of the tongue, 2 = 1/3–2/3 of the tongue, 3 = >2/3 of the tongue) and thickness (0 = none; 1 = thin, tongue papillae visible; 2 = thick, tongue papillae invisible) [29].

To assess intraexaminer reproducibility, 10% of subjects were reexamined. Cohen’s κ values for all clinical measurements (including dental caries status, gingival inflammation, periodontal status, plaque accumulation, and tongue coating evaluation) ranged from 0.87 to 0.98.

### Questionnaire

To supplement the oral examinations and obtain information about factors associated with halitosis, participants were asked to complete a 52-item self-administered questionnaire. This instrument has been used in halitosis clinics [30] and was modified and updated for the white-collar employee population. It was used to collect information about subjects’ sociodemographic characteristics (gender, age, education level, income), dietary habits (e.g., frequency of fruit, sweets, meat, and tea consumption; smoking and alcohol consumption habits), oral hygiene habits (toothbrushing frequency, use of dental floss and mouthwash, tongue scraping), and oral discomfort (e.g., self-reported gingival inflammation and bleeding, oral ulcers, tongue coating, dry mouth, food impaction). Most questionnaire items concerned respondents’ habits in the previous month. Subjects were also asked to provide medical history data, including information about gastrointestinal diseases and discomfort.

### Statistical analysis

Descriptive statistics (means, standard deviations, and percentages) were calculated for subjects’ sociodemographic characteristics and VSC measurements. Chi-squared tests were performed to examine differences in VSC values [dichotomized as 0 (VSC < 110 ppb) and 1 (VSC ≥ 110 ppb)] according to clinical and lifestyle factors. Student’s *t*-test and one-way analysis of variance were used to examine differences in VSC values. Multiple factor analyses were performed to explore factors associated with oral malodor. VSC values (dichotomized as 0 and 1) were examined using multiple logistic regression, and continuous VSC values were examined using analysis of covariance. All variables with *P* values < 0.2 in bivariate analyses were entered into these two regression models. All statistical analyses were conducted using SPSS software (ver. 20.0; IBM Corporation, Armonk, NY, USA), with the significance level set to *P* < 0.05.

## Results

### Study participants

Of the 805 white-collar workers invited to participate in the study, 720 [347 men, 373 women; mean age, 30.4 (range, 22–70) years] were enrolled (89.4% response rate). Subjects who were absent on the examination days due to business trips or illness and those unwilling to undergo dental examination were excluded from the study. Clinical and survey data from the 720 subjects were included in the final analyses. No difference in age or gender distribution was noted between subjects who did and did not participate in the study.

### Oral malodor and general characteristics

The mean VSC value was 117 ± 103 ppb. VSC values exceeded 110 ppb, the cutoff value for halitosis, in 33.2% of subjects. Halitosis was more prevalent in men than in women (*P* < 0.01). VSC values were higher in participants with higher body mass indices (BMIs) (*P* < 0.05). No significant difference in the prevalence of halitosis according to participants’ place of birth, education level, or monthly income was observed (Table 1).

**Table 1:** Relationships between oral malodor measurements and sociodemographic and background characteristics of white-collar employees in China^#^.

	*n*	% with VSC ≥ 110 ppb	*P*	VSC value (mean ± SD)	*P*
Age (years)			0. 420		0.190
21–30	479	40.3		111 ± 97	
31–40	179	39.1		136 ± 120	
>40	62	40.3		115 ± 82	
Gender			0.003		0.002
Male	347	38.6		130 ± 117	
Female	373	28.2		106 ± 86	
Place of birth			0.941		0.304
Shanghai region	387	33.1		114 ± 96	
Other	333	33.3		122 ± 110	
BMI (kg/m^2^)*			0.170		0.015
<18	52	30.8		111 ± 101	
18–24	521	31.7		112 ± 96	
>24	143	39.9		140 ± 124	
Education level*			0.067		0.841
High school or less	154	39.6		121 ± 83	
Undergraduate	404	33.2		118 ± 101	
Master's degree or more	158	27.2		114 ± 122	
Monthly income (RMB)*			0.773		0.539
0–3000	86	36.0		115 ± 83	
3001–6000	162	32.1		114 ± 93	
6001–9000	135	28.9		112 ± 97	
9001–12000	115	33.0		112 ± 85	
>12000	121	35.5		130 ± 120	

^#^Obtained by Chi-squared test, Student’s *t*-test (two groups), and one-way ANOVA.

*Some data are missing for these variables.

VSC: volatile sulfur compound; SD: standard deviation; BMI: body mass index; RMB: Ren Min Bi (name of the currency in China).

### Relationships between oral malodor and clinical and lifestyle factors

Relationships between oral malodor measures and clinical factors are shown in Table 2. Tongue coating area and thickness, as well as mean PPD, were associated positively with halitosis (*P* < 0.01). No significant association was found between oral malodor measures and DMFT or DMFS indices or GIs.

**Table 2:** Relationships between oral malodor measurements and clinical factors^#^.

	*n*	% with VSC ≥ 110 ppb	*P*	VSC value (mean ± SD)	*P*
Tongue coating thickness			0.007		0.003
None	344	27.6		106 ± 93	
Thin	260	36.9		122 ± 96	
Thick	116	41.4		142 ± 135	
Tongue coating area			0.005		0.009
None	342	27.2		105 ± 92	
≤1/3	80	36.3		124 ± 97	
1/3–2/3	154	35.7		123 ± 101	
≥2/3	144	43.1		138 ± 125	
Gingival index			0.091		0.143
<1	610	31.7		115 ± 100	
1–1.5	67	42.6		139 ± 134	
>1.5	43	44.2		135 ± 98	
Plaque index			0.251		0.629
<2	593	32.7		117 ± 106	
2–3	85	40.0		124 ± 94	
>3	42	26.2		105 ± 66	
Periodontal probing depth			<0.001		<0.001
<3.5 mm	682	31.7		114 ± 98	
≥3.5 mm	38	60.5		175± 153	

^#^Obtained by Chi-squared test, Student’s *t*-test (two groups), and one-way ANOVA.

VSC: volatile sulfur compound; SD: standard deviation.

No oral hygiene habit was related to oral malodor (Table 3). The percentage of subjects with VSCs ≥ 110 ppb was associated with eating within 2 hours prior to oral malodor measurement (*P* < 0.01), fruit intake frequency (*P* = 0.03), and work stress (*P* = 0.043; Table 4). VSC values were lower in subjects who had eaten within 2 hours prior to assessment (*P* < 0.01) and those with more frequent intakes of fruits, sweet foods, and tea (*P* < 0.05; Table 4). No significant association between oral malodor measures and self-reported oral or systemic diseases or oral discomfort was observed.

**Table 3:** Relationships between oral malodor measurements and oral hygiene habits^#^.

	*n*	% with VSC ≥ 110 ppb	*P*	VSC value (mean ± SD)	*P*
Brushing < 2 hours before examination			0.649		0.278
Yes	24	37.5		140 ± 126	
No	696	33.0		117 ± 102	
Toothbrushing frequency*			0.930		0.961
≥2×/day	568	32.9		117 ± 103	
1×/day	145	33.8		119 ± 101	
Never or seldom	5	40.0		120 ± 60	
Dental floss use*			0.934		0.357
Yes	116	33.6		110 ± 71	
No	602	33.2		119 ± 108	
Mouthwash use			0.115		0.199
Yes	236	29.2		110 ± 99	
No	484	35.1		121 ± 104	
Gum chewing*			0.212		0.992
Yes	506	31.8		117 ± 106	
No	213	36.6		118 ± 95	
Tongue scraping			0.518		0.192
Yes	210	31.4		110 ± 86	
No	510	33.9		121 ± 108	

^#^Obtained by Chi-squared test, Student’s *t*-test (two groups), and one-way ANOVA.

*Some data are missing for these variables.

VSC: volatile sulfur compound; SD: standard deviation.

**Table 4:** Relationships between oral malodor measurements and health behaviors^#^.

	*n*	% with VSC≥ 110 ppb	*P*	VSC value (mean ± SD)	*P*
Food intake < 2 hours before examination*			<0.001		<0.001
Yes	407	24.3		96 ± 71	
No	312	44.9		146 ± 128	
Fruit intake			0.031		0.046
≥4×/week	482	30.7		113 ± 99	
≤3×/week	164	34.8		120 ± 103	
Seldom/never	74	45.9		144 ± 121	
Sweet food intake*			0.202		<0.001
≥4×/week	269	31.6		106 ± 77	
≤3×/week	369	32.5		117 ± 96	
Seldom/never	81	42.0		160 ± 172	
Pungent food intake*			0.145		0.418
≥4×/week	260	30.4		113 ± 102	
≤3×/week	228	31.1		116 ± 106	
Seldom/never	231	38.1		125 ± 101	
Seafood intake			0.398		0.333
≥4×/week	127	31.5		112 ± 96	
≤3×/week	236	30.5		112 ± 100	
Seldom/never	357	35.6		123 ± 106	
Fried food intake*			0.992		0.790
≥4×/week	215	35.5		115 ± 99	
≤3×/week	261	33.0		117 ± 102	
Seldom/never	243	33.3		121 ± 106	
Meat intake*			0.136		0.618
≥4×/week	483	31.3		115 ± 102	
≤3×/week	120	40.8		123 ± 96	
Seldom/never	115	33.9		123 ± 113	
Vegetable intake			0.610		0.388
≥4×/week	597	32.7		115 ± 96	
≤3×/week	86	33.7		129 ± 140	
Seldom/never	37	40.5		127 ± 104	
Tea intake			0.082		0.003
≥4×/week	411	30.2		106 ± 80	
≤3×/week	124	33.9		127 ± 122	
Seldom/never	185	39.5		135 ± 127	
Work stress			0.043		0.231
Heavy	255	52.4		111 ± 46	
Medium	400	36.8		127 ± 118	
Low	65	30.5		113 ± 96	
Regular physical activity*			0.132		0.545
Yes	361	30.5		120 ± 113	
No	358	35.8		115 ± 91	
Current smoker*			0.568		0.287
Yes	89	36.0		128 ± 115	
No	629	32.9		116 ± 101	
Current alcohol consumption*			0.678		0.604
Yes	174	34.5		121 ± 107	
No	543	32.8		117 ± 101	

^#^Obtained by Chi-squared test, Student’s *t*-test (two groups), and one-way ANOVA.

*Some data are missing for these variables.

VSC: volatile sulfur compound; SD: standard deviation.

Regression models indicated that the percentage of subjects with VSCs ≥ 110 ppb was associated significantly with tongue coating thickness, PPD, and no food consumption within 2 hours prior to assessment (Table 5). VSC values were associated significantly with tongue coating thickness, PPD, no food consumption within 2 hours prior to assessment, and less frequent intake of sweet foods (Table 6).

**Table 5:** Multiple logistic regression model for VSC values (0: <110 ppb, 1: ≥110 ppb) ^#^.

	Odds ratio	95% CI	*P*
Tongue coating thickness			0.005
None^a^			
Thin	1.60	1.11–2.28	
Thick	1.90	1.21–3.00	
PPD			0.003
<3.5 mm^a^			
≥3.5 mm	2.90	1.45–5.80	
Food consumption < 2 hours before examination			<0.001
No^a^			
Yes	0.40	0.29–0.55	
(Intercept)	0.012		<0.001

^#^Obtained by multiple logistic regression.

^a^Reference group.

CI: confidence interval; PPD: periodontal probing depth.

**Table 6:** Analysis of covariance model for VSC values as continuous variable^#^.

	*F*	*B*	*P*
Tongue coating thickness	6.026		0.003
None^a^		0	
Thin		18.955	
Thick		35.242	
PPD	8.185		0.004
<3.5 mm^a^		0	
≥3.5 mm		46.604	
Food consumption <2 hours before examination	43.558		<0.001
Yes^a^		0	
No		48.385	
Intake frequency of sweet food	9.587		<0.001
≥4×/week ^a^		0	
≤3×/week		45.706	
Seldom/never		55.071	
(Intercept)	308.004		<0.001

^#^Obtained by analysis of covariance.

^a^Reference group.

PPD: periodontal probing depth.

## Discussion

This is the first study to evaluate the prevalence and factors related to halitosis in the specific population of white-collar employees in Shanghai, China. More than one-third (33.2%) of VSC values in the study population exceeded 110 ppb, similar to the results of previous studies [16,17,19]. Although organoleptic methods are the gold standard for oral malodor assessment, we only measured VSC levels with the Halimeter monitor. As a portable sulfur detector, the Halimeter provides useful data for clinical studies of halitosis [31]. Omitting organoleptic methods also simplified the examination procedure and saved time. However, this omission was a definite limitation of this study, and future studies should include both measurements.

In the present study, more men than women had VSCs ≥ 110 ppb and higher VSC values in bivariate analysis, consistent with the results of a study conducted in Brazil [14]. This finding may be explained by better oral knowledge, attitude, and oral hygiene among women [32,33]. In the present study, gender was not associated with halitosis in regression analyses adjusted for confounding factors. In contrast, halitosis was found to be more prevalent among women than men in a study conducted in Kuwait. As a self-reported questionnaire was used to identify halitosis in that study, the results may reflect women’s overestimation of their oral malodor status [11]. Most epidemiological studies of halitosis, including a large-scale study conducted in China in 2002, have found no association between oral malodor and gender [12,15,16,19,20]. These discrepancies in study findings may due to differences in diagnostic methods and study populations. The association between gender and halitosis should be investigated further.

The finding that VSC values were higher in subjects with BMIs > 24 in bivariate analysis is similar to the results of a study conducted in Israel [15]. High BMI has been found to be associated with many diseases, such as diabetes, hypertension, sleep apnea, and periodontitis [34,35]. Sleep apnea may cause dry mouth, a risk factor for oral malodor [36].

The dorsum of the tongue has long been considered the most important site for the development of halitosis, as abundant desquamated cells and leukocytes are retained and anaerobic bacteria grow on its irregular surface [6,8,16,37–40]. Although tongue coating thickness and area were associated with halitosis in bivariate analysis, only thickness remained significantly associated with halitosis after adjusting for confounding factors in the present study. Thus, the content, rather than extent, of tongue coating may contribute to malodor production. We also found that eating within 2 hours prior to examination dramatically reduced the percentage of subjects with VSCs ≥ 110 ppb and VSC values, perhaps because of the mechanical cleansing (“scraping”) of the tongue surface by food [41].

Several studies have indicated that periodontal disease is also a source of halitosis [12, 42–46]. In adjusted analyses, we found that PPD was significantly associated with halitosis. In a recent study, halitosis was more likely to be detected in subjects with periodontitis (odds ratio = 9.2) [46]. However, some researchers did not find association between halitosis and periodontitis and periodontal pocket is considered a “closed” environment that only a small fraction of sulfur compounds escape into the oral cavity [47,48]. Periodontal pathogenic bacteria, such as *Porphyromonas gingivalis*, *Tannerella forsythia*, and *Prevotella intermedia*, have been shown to contribute to VSC production [49–51]. A recent study demonstrated that the amount of *P*.*gingivalis* residing on the tongue dorsum may play a key role in oral malodor production in periodontitis patients [46]. So the reason for the significant relationship between PPD and oral malodor may not contribute to the periodontal pocket, but to the periodontal pathogenic bacteria especially the bacteria residing on tongue dorsum. In the present study, no radiographic assessment of bone loss was performed and the increased pocket levels may be attributed to gingival hyperplasia and pseudo-pocketing in absence of clinical attachment level measurements, the collected data may not reflect the true periodontal situation. This limitation should be avoided in future studies.

In analyses controlling for confounding factors, such as age and gender, we found that more frequent intake of sweet foods reduced VSC concentrations. The presence of carbohydrates, such as glucose and sucrose, has been reported to inhibit the expression and activity of trypsin-like enzyme, which is capable of degrading peptides that may produce malodorous compounds by producing an acidic environment [52–54]. Another study showed that 10 children with moderate to high caries activity who were more likely to consume sugar-containing snacks habitually were free from halitosis [55]. The present study may be the first epidemiological investigation to reveal that the consumption of sweet foods can inhibit VSC production. Fruit consumption was also associated with halitosis in bivariate analysis. As halitosis originates in the oral cavity as a result of proteolytic degradation by anaerobic Gram-negative oral bacteria of various sulfur-containing substrates [2,7], we assume that a greater frequency of fruit intake was accompanied by reduced protein consumption, resulting in less VSC production. Furthermore, as most fruits are sweet, the mechanism of halitosis inhibition may be similar to that of sweet food. Further large-scale epidemiological studies are needed to explore associations between diet and halitosis.

Tea is a very popular beverage in China. Green tea has been reported to reduce VSC levels immediately after consumption *in vivo* and *in vitro* [56]. The inhibition of halitosis may due to the antibacterial effect of green tea and suppression of the mgl gene, which encodes L-methionine-α-deamino-γ-mercaptomethane-lyase (METase), an enzyme producing methyl mercaptan [57,58]. In the present study, more frequent tea intake was related to lower VSC levels in bivariate analysis, but not in the regression analysis adjusted for confounding factors. We did not ask participants about the type and timing of tea consumption, which limited our ability to explore this association. The relationship between tea consumption and halitosis should be investigated further.

More than 90% of the subjects in this study complained of moderate to severe work stress. A greater proportion of participants with heavy work stress had VSCs ≥ 110 ppb in the bivariate analysis. One animal experiment showed that rats under stress produced more VSCs [59]. In a recent study, stress was shown to increase the production of VSCs, especially hydrogen sulfide, in men and women [60]. However, work stress was not included in the final regression model in the present study. Thus, the relationship between work stress and halitosis requires further evaluation.

This study was an epidemiological study of halitosis in a specific population rather than the general population. Thus, the results may only be applicable to individuals with similar backgrounds and working conditions. Caution should be taken when seeking to apply the results to other populations.

## Conclusion

In the present study, tongue coating thickness and PPD were significantly related to measures of oral malodor. As most cases of halitosis originate from the oral cavity, tongue cleaning and periodontal health maintenance may help to reduce oral malodor. The results of our study suggest that appropriate intake of sweet foods, while controlling for the risk of caries development, can reduce VSC production. However, the relationship between halitosis and sweet food consumption, as well as the appropriate amount of such foods to control VSC production without causing caries, requires further investigation.

## Supporting Information

S1 STROBE Checklist STROBE Checklist cross-sectional. (DOC)
